# Dual detection and novel genotypes of *Giardia* and *Leishmania* in *Ochotona curzoniae* from Zoige County, Qinghai-Tibet Plateau

**DOI:** 10.3389/fvets.2026.1855828

**Published:** 2026-06-22

**Authors:** Lu Deng, Nian-Chun Yin, Zhun Yi, Ri-hong Jike, Hong-Xi Chen, Tian-Cai Tang, Li-Li Hao

**Affiliations:** 1 College of Culinary and Food Science Engineering, Sichuan Tourism University, Chengdu, China; 2 Animal Disease Prevention and Control Center of the Suining, Suining, China; 3 Aba Prefecture Agricultural Science Research Institute, Aba, China; 4 Agricultural and Rural Bureau of Liangshan Yi Autonomous Prefecture of Sichuan Province, Xichang, China; 5 College of Animal Husbandry and Veterinary Medicine, Southwest Minzu University, Chengdu, China; 6 Faculty of Agriculture, Forestry and Food Engineering, Yibin University, Yibin, China

**Keywords:** *Leishmania*, genotypes, Zoige County, *Giardia lamblia*, Plateau pika

## Abstract

**Introduction:**

*Giardia* and *Leishmania* are important zoonotic parasites, yet their transmission dynamics in wildlife hosts on the Qinghai-Tibet Plateau remain unclear. As a keystone species on the Plateau, *Ochotona curzoniaes* overlap spatially with livestock and human activity areas and may serve as potential reservoir hosts for these pathogens. However, relevant molecular epidemiological data are currently lacking.

**Methods:**

To address this knowledge gap, the present study investigated the prevalence and molecular characteristics of *Giardia* and *Leishmania* in 114 *O. curzoniaes* collected from five townships in Zoige County, China, between March and December 2023. Intestinal contents and spleen samples were analyzed using nested PCR targeting *Giardia bg*, *gdh*, *tpi* genes and *Leishmania ITS-1* gene. Positive products were sequenced, followed by BLAST comparison and phylogenetic analysis for species identification and genotyping.

**Results:**

Results showed 50 samples were *Giardia*-positive (43.9%), including 44 *bg*-positive and 14 *gdh*-positive samples, with eight samples positive for both genes. Five samples (4.39%) were positive for *Leishmania*. Significant differences in *Giardia* prevalence among the five locations were observed, whereas *Leishmania* prevalence did not differ significantly. Phylogenetic analysis identified *G. intestinalis* assemblage E (*n* = 38), *G. microti* (*n* = 6) based on *bg*, and a potentially novel genotype, tentatively designated *Giardia* sp. clone Z5 (*n* = 14) based on *gdh*. All *Leishmania* isolates were identified as *L. major* (*n* = 5).

**Discussion:**

This study provides the first evidence of *G. intestinalis* assemblage E, *G. microti*, *Giardia* sp. clone Z5, and *L. major* in *O. curzoniae*, highlighting their role as potential wildlife reservoirs and supporting ongoing surveillance and genotyping of zoonotic protozoa in the Qinghai-Tibet Plateau.

## 1 Introduction

*Giardia* is a zoonotic intestinal protozoan with a global distribution. It undergoes a two-stage life cycle, alternating between the cyst and trophozoite stages, and infects a broad range of hosts including humans, domestic animals, and wildlife (1). Infection can lead to giardiasis, a clinical condition characterized by diarrhea, abdominal pain and bloating. Globally, symptomatic giardiasis is estimated to cause approximately 280 million human cases annually (2). The prevalence of this disease exhibits significant geographical variation, affecting an estimated 2–5% of populations in developed countries, compared to 20–30% in developing nations (3, 4). The prevalence of *Giardia* varies substantially among animal hosts worldwide and is commonly strongly associated with age and husbandry density (5). In livestock, overall prevalence in bovines ranges from 1.09 to 74.2%, with calves and dairy cattle significantly higher than beef cattle (6, 7); in sheep, prevalence ranges from 1.5 to 89.2%, and lambs may have up to a sevenfold higher risk than adults (5); in goats, 4.0–43.5% (5, 8); and in pigs, 3.5–31.1% (5, 8). In companion animals, dogs and cats show prevalences of 1.1–45.9%, with particularly high rates in juveniles and high-density settings (e.g., kennels and shelters). In wildlife, *Giardia* has also been detected in pinnipeds, lagomorphs, reptiles, wild canids and felids (0.6–41.0%) (5, 8, 9). The epidemiology of *Giardia* is complicated by its substantial genetic diversity. The seven recognized species of *Giardia* include *G. intestinalis* (syn. *G. lamblia*, *G. duodenalis*), which infects humans and other mammals and is classified into eight distinct assemblages (A–H) that differ in their host specificity (10–12). Among these, assemblages A and B are considered zoonotic, with a broad host range encompassing humans, non-human primates, dogs, cats, and wildlife (8). In contrast, assemblages C–H exhibit greater host restriction: C and D primarily infect canids, while E, F, G, and H predominantly infect artiodactyls, cats, rodents, and marine mammals, respectively (8, 13–15). Although research on *Giardia* infections in animals has increased, most efforts have primarily focused on domestic species and urban wildlife. Consequently, data on *Giardia* in wildlife remain scarce, particularly in ecologically unique regions such as the Qinghai-Tibet Plateau. Therefore, prevention and control in livestock and companion animals can be implemented effectively based on host species, age, husbandry/management practices, and common *Giardia* assemblages. However, data remain scarce on the population genetic structure, cross-species transmission potential, and local epidemiology of *Giardia* in wildlife (especially in high-altitude regions).

Leishmaniasis, caused by *Leishmania* spp., is a neglected zoonosis transmitted by sandflies, presenting as visceral, cutaneous, or mucocutaneous forms, with visceral *leishmaniasis* being highly fatal if untreated (16–18). In nature, *Leishmania* maintains its life cycle through mammalian reservoir hosts and vectors. Dogs have long been considered as the main reservoir host, but recent studies in wild rabbits and hares in Spain, Brazil, and Israel revealed a “rabbit–sandfly” transmission cycle (19–21). Despite these advances, data on *Leishmania* infection in leporids within China remain strikingly scarce.

The Plateau pika (*Ochotona curzoniae*) is a small lagomorph abundant on the Qinghai-Tibet Plateau, often hosting multiple zoonotic pathogens including *Bartonella* (21.7%) (22), *Echinococcus multilocularis* (6.02%) (23), *Toxoplasma gondii* (3.96%) (24), and *Cryptosporidium* (7.0%) (25). In Zoige County, *O. curzoniae* live in close proximity to livestock and human settlements, creating frequent opportunities for cross-species transmission. While *Cryptosporidium* has been reported in local pikas (25), the occurrence of *Giardia* and *Leishmani*a in this keystone species remains entirely unexplored. This study presents the first molecular investigation of *Giardia* and *Leishmania* in *O. curzoniae*, aiming to determine their prevalence and genetic identity, with implications for safeguarding public health and livestock productivity on the Plateau.

## 2 Materials and methods

### 2.1 Sample collection

Between March and December 2023, 114 *O. curzoniae* were captured using live traps from five townships in Zoige County (Dazha Temple 24, Axi 20, Hongxing 20, Maixi 20, Tangke 30). To minimize suffering, *O. curzoniae* were first anesthetized with isoflurane and subsequently euthanized by cervical dislocation. Intestinal contents (*Giardia* detection) and spleens (*Leishmania* detection) samples were collected using sterile gloves, stored in liquid nitrogen, transported to the laboratory, and stored at −80 °C. The body of each *O. curzoniae* was deeply buried to avoid being eaten by dogs, cats, or other wild carnivores.

### 2.2 DNA extraction

Approximately 150 mg of intestinal and spleen tissue was homogenized in ddH_2_O and centrifuged at 5,000 × *g* for 5 min. DNA was extracted from the intestinal contents and spleen tissues of *O. curzoniae* using the TIANamp Stool DNA Kit (Cat. No. DP328) and TIANamp Genomic DNA Kit (Cat. No. DP304) (TIANGEN Biotech Co., Ltd., Beijing, China)^1^ (accessed on 20 December 2023), respectively, according to the manufacturer’s instructions. DNA concentration and purity were measured with a NanoDrop 2000 (Thermo Fisher, United States), selecting samples >30 ng/μL (A260) for subsequent experiments. Extracted DNA was stored at −20 °C.

### 2.3 PCR amplification

Nested PCR was performed targeting *Giardia bg* (26), *gdh* (27), *tpi* (26), and *Leishmania ITS-1* (28) genes. Primer details are listed in Table 1. PCR reactions (25 μL) included 22 μL T3 Super PCR Mix (Qingke Bio), 1 μL of each primer (10 μmol. L^−1^), and 1 μL DNA template. Positive (*G. intestinalis* and *Leishmania* DNA) and negative (ddH_2_O) controls were included. PCR cycling: 98 °C 2 min; 35 cycles of 98 °C 10 s, 57 °C 10 s, 72 °C extension (size-dependent); final extension 72 °C 8 min; hold 16 °C. Products were visualized by 1.3% agarose gel electrophoresis.

**Table 1:** Primer sequences used for *Giardia* and *Leishmania* identification.

Species	Target gene	Primer name	Primer sequence (5′–3′)	Product (bp)	Annealing temperature(°C)	References
*Giardia* sp.	*bg*	BgF1	AAGCCCGACGACCTCACCCGCAGTGC	753	55	26
		BgR1	GAGGCCGCCCTGGATCTTCGAGACGAC			
		BgF2	GAACGAACGAGATCGAGGTCCG	511	55	
		BgR2	CTCGACGAGCTTCGTGTT			
	*gdh*	GdhF1	TTCCGTRTYCAGTACAACTC	754	50	27
		GdhR1	ACCTCGTTCTGRGTGGCGCA			
		GdhF2	ATGACYGAGCTYCAGAGGCACGT	530	60	
		GdhR2	GTGGCGCARGGCATGATGCA			
*Leishmania* sp.	ITS	ITSF1	CTGGATCATTTTCCGATG	763	55	28
		ITSR1	TGATACCACTTATCGCACTT			
		ITSF2	CATTTTCCGATGATTACACC	587	55	
		ITSR2	TACTGCGTTCTTCAACGA			

### 2.4 Sequence analysis and phylogenetic tree

The final positive PCR products of *Giardia* and *Leishmania* were subjected to Sanger bidirectional sequencing by Chengdu Branch of Sangon Biotech Company (Shanghai, China) using the second pair of primers. Firstly, all the obtained sequences were analyzed and manually edited by employing DNA Star and were subjected to nucleotide BLAST search through the NCBI database. Subsequently, the sequences of *Giardia* and *Leishmania* with the highest similarity to the blast results were selected (1–4 sequences), and the bg and gdh gene sequences of *Giardia*, including *G. lamblia* assemblage A-H, *G. agilis*, *G. ardeae*, *G. psittaci*, *G. muris*, *G. microti*, *G.* var*ani*, and the ITS gene sequences of *Leishmania*, including *L. infantum*, *L. donovani*, *L. tropica*, *L. major*, *L. mexicana*, *L. braziliensis*, were selected as reference sequences to construct the phylogenetic trees of *Giardia* and *Leishmania*, respectively. Lastly, the phylogenetic tree was constructed based on the Neighbor-Joining (NJ) method using MEGA 11.0, and 1,000 replicates (bootstrap value) were selected to assess the robustness of the findings.

### 2.5 Statistical analysis

Firstly, Pearson Chi-square test with the software SPSS 27.0 was used to assess whether a significant difference in prevalence of *Giardia* and *Leishmania* infections among *O. curzoniae* across sampling locations. A *p* < 0.05 was considered significant. Secondly, the specimen numbers analyzed in the present study are relatively low (when the count in any expected cell was <5), therefore the statistical outcomes obtained where reanalyzed with Fisher’s exact test and confirmed (29).

## 3 Results

### 3.1 Phylogenetic analysis based on bg and gdh gene of *Giardia*

**Figure 1:**
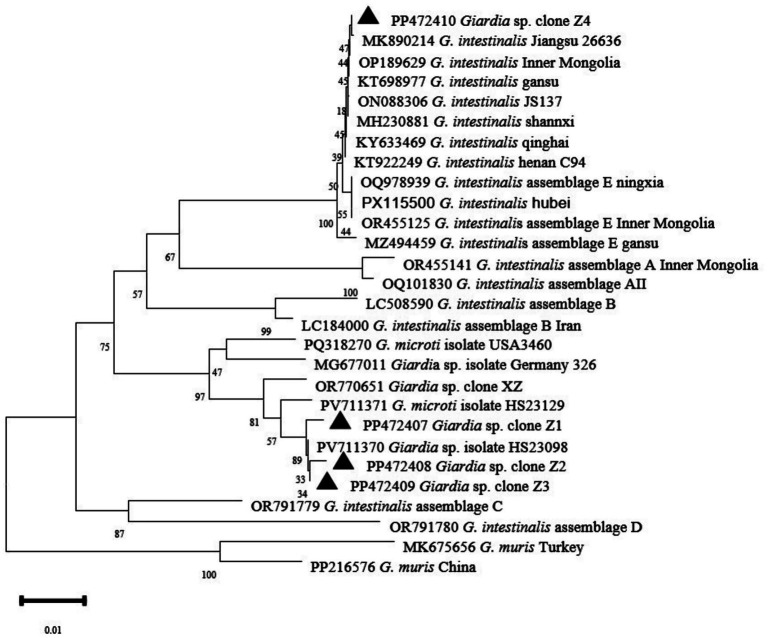
Phylogenetic tree based on *bg* gene of *Giardia*. ▲: Sequence obtained from this study. Bootstrap values from 1,000 pseudoreplicates are indicated at the left of the supported node. Scale bar indicates an evolutionary distance of 0.01 substitutions per site in the sequence.

Positive *bg* and *gdh* gene products of *Giardi*a were sequenced, and five distinct sequences were obtained (GenBank accession numbers: PP472407–PP472410, PP502972). Phylogenetic analysis revealed two well-defined clades based on *bg* gene sequences (Figure 1). *Giardia* sp. clones Z1–Z3 (PP472407–PP472409) clustered with *Giardia* sp. isolate HS23098 (PV711370), *G. microti* isolate HS23129 (PV711371), and *Giardia* sp. clone XZ (OR770651) from China, showing the closest relationship with sequence identities of 99–100%. *Giardia* sp. clone Z4 (PP472410) clustered with *G. intestinalis* isolates from Jiangsu (MK890214), Inner Mongolia (OP189629), Gansu (KT698977), Shaanxi (MH230881), Qinghai (KY633469), and Hubei (PX115500), and further grouped with *G. intestinalis* assemblage E isolates from Ningxia (OQ978939), Inner Mongolia (OR455125), and Gansu (MZ494459), sharing 100% sequence identity.

**Figure 2:**
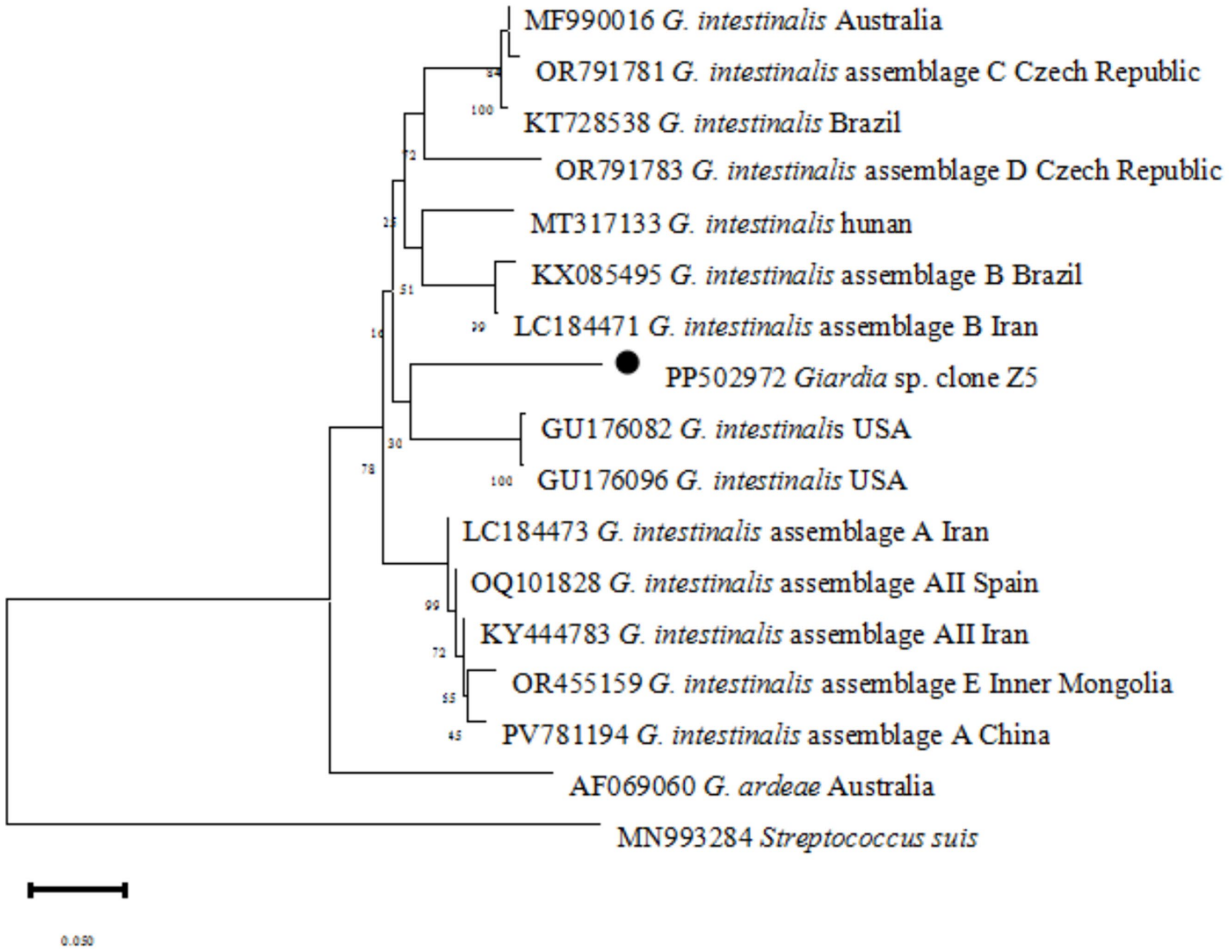
Phylogenetic tree based on *gdh* gene of *Giardia*. ●: Sequence obtained from this study. Bootstrap values from 1,000 pseudoreplicates are indicated at the left of the supported node. Scale bar indicates an evolutionary distance of 0.05 substitutions per site in the sequence.

Notably, *Giardia* sp. clone Z5 (PP502972), based on the *gdh* gene, formed an independent branch in the phylogenetic tree (Figure 2). It was closely related to *G. intestinalis* sequences (GU176082, GU176096) from seals in the United States but shared only 84.37% nucleotide identity. This genetic distance suggests that the isolate may represent a potentially novel genotype.

### 3.2 Phylogenetic analysis based on ITS gene of *Leishmania*

**Figure 3:**
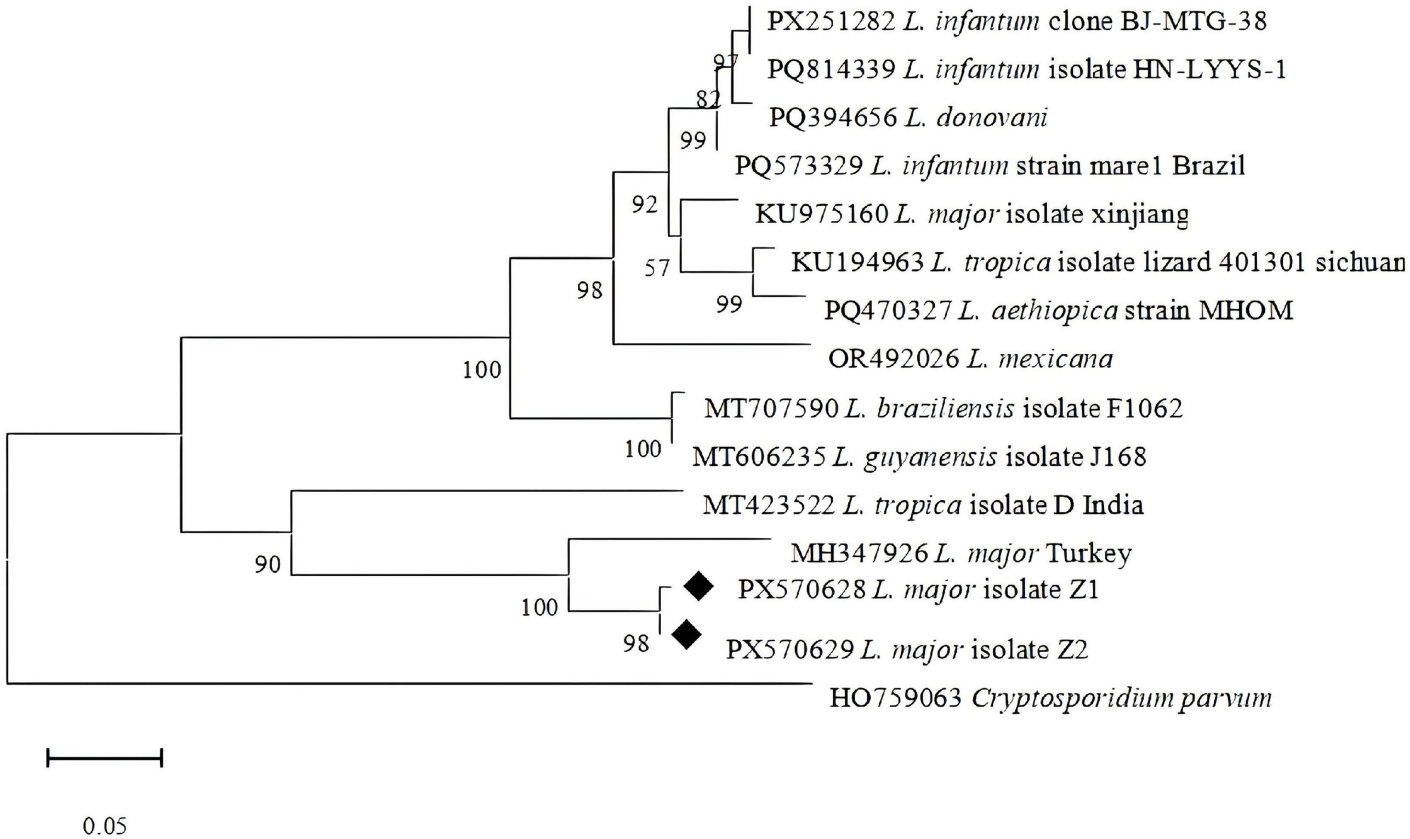
Phylogenetic tree based on *ITS-1* gene of *Leishmania*. ♦: Sequence obtained from this study. Bootstrap values from 1,000 pseudoreplicates are indicated at the left of the supported node. Scale bar indicates an evolutionary distance of 0.05 substitutions per site in the sequence.

Two *ITS-1* gene sequences of *Leishmania* were obtained from positive samples (GenBank PX570628 and PX570629). Both were identified as *Leishmania major*, designated as *L. major* isolate Z1 and *L. major* isolate Z2. Phylogenetic analysis showed that these isolates clustered with *L. major* from Turkey (MH347926), exhibiting the closest genetic relationship with 98.43% sequence identity (Figure 3).

### 3.3 Detection of *Giardia* and *Leishmania* in *O. curzoniae*

Among 114 samples of *O. curzoniae*, a total of 53 were positive for *Giardia* or *Leishmania*, yielding an overall positivity rate of 46.49%. *Giardia* was detected in 50 samples (43.86%), including 44 positive for the *bg* gene (38.6%), 14 for the *gdh* gene (12.28%), and 8 positive for both genes (7.02%). The *tpi* gene remained undetectable despite multiple attempts. *Leishmania* was detected in five samples (4.39%), with mixed infections of *Giardia* and *Leishmania* observed in two samples (1.75%).

The detection rate of *Giardia* varied significantly among sampling sites (Pearson and Fisher tests, *p* < 0.001). The highest prevalence was recorded in Maixi (100%, *n* = 10), followed by Hongxing (70.0%), Tangke (46.7%), Axi (10%), and none detected in Dazhasi. *bg* gene sequencing identified *G. intestinalis* assemblage E (*n* = 38) and *G. microti* (*n* = 6), while *gdh* sequences were all identified as *Giardia* sp. clone Z5 (*n* = 14).

The detection rate of *Leishmania* showed no significant difference among sites (*p* > 0.05), with the highest rate observed in Dazhasi (8.33%, *n* = 2), followed by Axi and Maixi (5.0%, *n* = 1 each), Tangke (3.33%), and none in Hongxing (Table 2).

**Table 2:** Prevalence and species of *Giardia* and *Leishmania* in *O. curzoniae.*

Variable	Overall infection rate (%) (no. Positive/no. of samples)	Infection rate (%) (n)		Species		
		*Giardia* spp.	*Leishmania* spp.	*Giardia* spp.		*Leishmania* spp.
				*bg*	*gdh*	
Sampling sites						
Dazhasi	8.33 (2/24)	0	8.33 (2)			*L. major* (2)
Axi	15.0 (3/20)	10.0 (2)	5.0 (1)	*G. intestinalis* (2)		*L. major* (1)
Hongxing	70.0 (14/20)	70.0 (14)	0	*G. intestinalis* (13)	*Giardia* sp. clone Z5 (3)	
Maixi	100.0 (20/20)	100.0 (20)	5.0 (1)	*G. intestinalis* (13), G*. microti* (3)	*Giardia* sp. clone Z5 (9)	*L. major* (1)
Tangke	46.67 (14/30)	46.67 (14)	3.33 (1)	*G. intestinalis* (10)*, G. microti* (3)	*Giardia* sp. clone Z5 (2)	*L. major* (1)
Month						
3–5	23.07 (6/26)	23.07 (6)	0	*G. intestinalis* (5)	*Giardia* sp. clone Z5 (1)	
6–9	61.11 (33/54)	57.4 (31)	7.4 (4)	*G. intestinalis* (25), G*. microti* (5)	*Giardia* sp. clone Z5 (9)	*L. major* (4)
10–11	41.17 (14/34)	38.23 (13)	2.9 (1)	*G. intestinalis* (8), G*. microti* (1)	*Giardia* sp. clone Z5 (4)	*L. major* (1)
Total	46.49 (53/114)	43.86 (50)	4.39 (5)	*G. intestinalis* (38), G*. microti* (6)	*Giardia* sp. clone Z5(14)	*L. major* (5)

The overall detection rate from June to September (61.11%) was significantly higher than those observed from March to May (23.07%). Similarly, the detection rate of *Giardia* from June to September (57.4%) was significantly higher than those in March to May (23.07%) (*p* < 0.05), No statistically significant differences were found among the other groups (*p* > 0.05) (Table 2).

## 4 Discussion

This study reports the first detection of *Giardia* in *O. curzoniae* from Sichuan Province, with a notably high infection rate of 43.86% (50/114). This prevalence is significantly higher than those previously reported in other animal hosts within the province, including yaks (15.7%) (30), adult sheep (14.9%) (31), stray dogs (11.3%) (32), pet chipmunks (8.6%) (33), racehorses (8.3%) (34) and forest musk deer (2.24%) (35). Such a high infection rate may be closely associated with the unique ecological behavior of *O. curzoniae*. These animals are typical colonial burrowers, and their dense underground tunnel systems provide ideal conditions for fecal–oral transmission (8, 36). The relatively stable temperature and humidity inside burrows, combined with low ultraviolet radiation, significantly prolong the survival of *Giardia* cysts (37, 38). In contrast, although domestic livestock are also gregarious, the open nature of grazing environments exposes cysts to UV light and climatic fluctuations, thereby reducing transmission efficiency. Similarly, stray dogs, pet chipmunks, and racehorses are less likely to encounter heavily contaminated environments due to differences in mobility and human management. Furthermore, this study employed highly sensitive nested PCR, which can detect extremely low copy numbers of parasite DNA, including DNA from inactivated organisms or subclinical infections; this may be one reason for the relatively high detected prevalence. Because all samples were collected from the intestinal contents and spleens of wild *O. curzoniaes* in the field and preserved in liquid nitrogen, the conditions were not compatible with conventional microscopy, and therefore parallel microscopic examination was not performed. In subsequent surveys in surrounding areas, we will integrate molecular detection with microscopy to more accurately assess the epidemiological characteristics of *Giardia*.

Compared to global data on lagomorphs, the prevalence in this study falls between reports in Nigeria (72.3%) (39) and in Brazil (40.0%) (40), Algeria (29.5%) (41) and Spain (27.8%) (42). Within China, the prevalence in *O. curzoniae* is markedly higher than in Shaanxi (3.54%), Henan (8.4%), Xinjiang (1.9%), Shandong (11.2%), and Jilin and Liaoning (9.86%) (43–47). These findings indicate that the prevalence of *Giardia* in lagomorph hosts exhibits pronounced geographical variation, likely influenced by multiple factors including climate conditions, detection methodologies, host population density, and habitat characteristics.

At the molecular level, the positive rate of the *bg* gene (38.6%, 44/114) was significantly higher than that of the *gdh* gene (12.3%, 14/114), consistent with previous findings (30, 31, 39, 44, 45, 48) and reflecting differences in detection sensitivity among target genes. Feng et al. reported that most primer sets yield approximately 60% positivity for the *bg* gene but only 40–60% for *gdh* (8); likewise, a study on Ghanaian HIV patients showed that *bg* detection sensitivity (31.7%) was markedly higher than *gdh* (17.5%) (48), further supporting the superior diagnostic value of the *bg* gene. In addition, we repeatedly attempted to amplify the *tpi* gene in this study, but it remained undetected. Inconsistent multilocus genotyping results have been reported multiple times in *Giardia* studies. For example, Cacciò et al. and Lebbad et al. observed relatively high PCR failure rates for the *tpi* gene in human populations and isolates from multiple animal sources (27, 49). This is usually attributed to sequence variation in primer-binding regions of the *tpi* gene that reduces amplification efficiency (50), and is particularly common in atypical hosts (such as *O. curzoniaes*) or novel genetic lineages.

Genotyping based on the *bg* gene revealed the presence of both *G. microti* and *G. intestinalis* assemblage E in *O. curzoniae*, with the latter being dominant. Previous studies have detected assemblages A, B, and E in lagomorphs, with B being the most frequent (39–45, 47, 51); however, findings from Shandong Province align with our results, with assemblage E predominating (46). These observations suggest that geographical environment and host species may be key determinants shaping the distribution of *Giardia* assemblages. Notably, assemblage E typically infects artiodactyls and has been reported in yaks from Sichuan (30). Given that *O. curzoniae* and yaks share the same alpine grazing habitats in Zoige County, cross-species transmission may occur between these hosts. Moreover, assemblage E has been repeatedly identified in humans, particularly in children (52, 53), indicating its potential zoonotic risk. Therefore, it is essential to establish an integrated control strategy to disrupt the *Giardia* transmission chain among *O. curzoniae*, yaks, and humans.

Interestingly, eight samples exhibited inconsistent genotyping results between the *bg* and *gdh* loci, a phenomenon also reported in rabbits from Algeria and in human isolates from Iran and Egypt (8, 41, 54, 55). This inconsistency may result from mixed infections or allelic sequence heterozygosity (ASH). *Giardia* is a diplomonad parasite known to exhibit high ASH levels. Woschke et al. found ambiguous nucleotide positions in 20.9% of *tpi* sequences from assemblage B isolates (56), while Kooyman et al. further demonstrated that ASH levels in assemblages C and D are even higher than in B, and significantly greater than in A and E (57). Additionally, multi–host mixed infections have been widely reported (58, 59); Kareem et al. identified at least 25 mammalian species exhibiting *Giardia* coinfections (59). The sympatric distribution and frequent ecological interactions of *O. curzoniae* with yaks, Tibetan sheep, rodents, and canids in Zoige further increase the likelihood of mixed infections. Notably, some isolates in this study showed only 84.37% sequence identity to reference strains (GU176082, GU176096) in *gdh*-based analysis and were phylogenetically closest to assemblage B (Figure 2), suggesting a potentially novel genotype closely related to assemblage B, tentatively designated *Giardia* sp. clone Z5. This isolate shows relatively low homology to known Assemblage B, suggesting that it may represent an independent evolutionary lineage with substantial genetic divergence. This is similar to the previously observed inconsistency between locus polymorphism and genotyping in non-human primates (60) and human/macaque–derived isolates (27), further reflecting the evolutionary complexity and adaptive potential of this parasite. We speculate that this lineage may have arisen through long-term adaptive evolution within *O. curzoniae* hosts. Previous studies have shown that intestinal parasites, including *Giardia*, can adapt to specific gut microenvironments via genetic variation and host-imposed selective pressures (61). As a keystone species in alpine ecosystems, *O. curzoniaes* have unique physiological traits, gut microbiota composition, and geographic distribution patterns; these factors may jointly impose strong local selection pressures that drive genetic differentiation in *Giardia* populations parasitizing them, ultimately resulting in pronounced genetic distances. Future studies will expand the host range and incorporate analyses of additional genetic loci, or whole genome sequencing, to further clarify the taxonomic status of this isolate.

This study also represents the first detection of *Leishmania* spp. in *O. curzoniae* from Sichuan Province, with a prevalence of 4.39% (5/114), significantly lower than infection rates reported in local canine populations from Wenchuan (23.5%), Heishui (28.2%), and Jiuzhaigou (24.1%) (62). Molecular identification confirmed that the detected species was *L. major*, while the circulating strain in nearby dogs is *L. infantum*. Dogs are recognized as highly adapted reservoirs of *L. infantum*, capable of sustaining long-term infections due to their immunological tolerance (63, 64); in contrast, *L. major* is primarily maintained by rodent reservoirs (65). Studies have shown that some rodent hosts can carry *L. major* chronically without developing overt disease, thus acting as key reservoirs in transmission cycles (66). Given their ecological and behavioral similarities to these wild rodents, *O. curzoniae*–through their communal burrow systems–offer a favorable microenvironment for both sand fly breeding and parasite persistence, providing the ecological basis for their potential role as *L. major* reservoir hosts. However, their infection tolerance, parasite proliferation dynamics, and infection duration require further verification through controlled infection experiments.

The detection rate in this study is close to the reported prevalence in small wild rodents in Turkey (1.12%) and capybaras in Latin America (6.25%) (67, 68), but is markedly lower than that in great gerbils in central Iran (44.4%), rats (25%) and house mice (24%) in northern Greece (69, 70). These differences may be closely related to host ecological behavior, frequency of vector contact, and population density. *O. curzoniaes* inhabit burrow environments at higher elevations with colder climates, which may limit vector breeding and opportunities for contact, thereby reducing infection risk. In addition, species differ genetically and physiologically in their immune responses to *Leishmania*, which may lead to differences in susceptibility and pathogen load. For example, in the same area of Xinjiang, China, infection rates differed among livestock: sheep 30.36% (17/56), goats 21.57% (11/51), cattle 17.78% (8/45), and donkeys 21.62% (8/37) (71). Moreover, a systematic review reported that *Leishmania* has been detected in 189 wildlife species (72), indicating a broad host range. Although the prevalence in *O. curzoniaes* in this study was low, they may still act as one of the potential reservoir hosts in this ecosystem and, together with other wildlife and even livestock, contribute to the transmission of *Leishmania*.

The prevalence of *Giardia* (43.86%) was markedly higher than that of *Leishmania* (4.39%). This difference may be explained by the following factors: *Giardia* is transmitted directly via the fecal–oral route, and its environmentally resistant cysts can persist and spread rapidly within host populations through contaminated water sources or environments (73). Variations in environmental conditions and *O. curzoniaes* population density across different sampling sites may lead to differences in local transmission intensity. Moreover, *Giardia* cysts are capable of long-term survival and accumulation in cold and humid environments, potentially giving rise to localized transmission hotspots. These factors may together explain the significant differences in *Giardia* detection rates observed among sampling sites. In contrast, the life cycle of *Leishmania* depends strictly on specific sand fly vectors (74). Zoige County features a high–altitude cold climate, with an average elevation of 3,500 m and an annual mean temperature of 2–4 °C, along with distinctive vegetation (alpine swamp meadow) and soil types (peat/gley soil). These conditions are unsuitable for the breeding and activity of local sandfly vectors, resulting in a generally sparse distribution of sandflies. Consequently, the transmission cycle of *Leishmania* is severely restricted, which may account for both the low overall detection rate and the lack of significant differences in detection rates among sampling sites.

This study revealed that both the overall detection rate (61.11%) and the *Giardia* detection rate (57.4%) from June to September were significantly higher than those observed from March to May (23.07 and 23.07%, respectively), indicating a clear seasonal pattern. This seasonal variation may be related to the climatic conditions in Zoige County. The average temperature from June to September is 9 °C, whereas the average temperatures from March to May was below 3 °C. Such low temperatures may restrict both the activity of *O. curzoniae* and the transmission of *Giardia*.

In summary, this study highlights the dual ecological significance of *O. curzoniae* on the Qinghai-Tibet Plateau–as both ecological keystone species and potential reservoir hosts of zoonotic pathogens. Their unique social behavior and habitat ecology not only influence parasite transmission dynamics but may also position them as critical ecological bridges in the circulation of zoonotic diseases within the Plateau ecosystem.

## 5 Conclusion

Here, we report the first molecular evidence for the co-circulation of *Giardia* and *Leishmania* in *O. curzoniae* from the Zoige County of China. The prevalence was substantial for *Giardia* (43.9%) in contrast to a much lower prevalence for *Leishmania* (4.39%). Molecular characterization identified three distinct *Giardia* taxa: *G. intestinalis* assemblage E, *G. microti* and tentatively designated *Giardia* sp. clone Z5. A single *Leishmania* species (*L. major*) was detected. Collectively, our findings significantly expand the known repertoire of parasites harbored by *O. curzoniae* and highlight their potential role as a wildlife reservoir for zoonotic transmission in the region. Future investigations are warranted to determine the spatial distribution and zoonotic potential of these parasite genotypes in pika populations across the Qinghai-Tibet Plateau, which is essential for assessing the emerging risks to public health.

## Data Availability

The sequences generated in this study were submitted to the GenBank, The names of the repository/repositories and accession number(s) can be found at: https://www.ncbi.nlm.nih.gov/genbank/, PP472407–PP472410, PP502972, PX570628, and PX570629.

## Footnotes

1
https://en.tiangen.com/
